# Widespread Decoding of Tactile Input Patterns Among Thalamic Neurons

**DOI:** 10.3389/fnsys.2021.640085

**Published:** 2021-02-16

**Authors:** Anders Wahlbom, Jonas M. D. Enander, Henrik Jörntell

**Affiliations:** Neural Basis of Sensorimotor Control, Department of Experimental Medical Science, Lund University, Lund, Sweden

**Keywords:** thalamus, neurophysiology, tactile, information processing, integrative neurophysiology

## Abstract

Whereas, there is data to support that cuneothalamic projections predominantly reach a topographically confined volume of the rat thalamus, the ventroposterior lateral (VPL) nucleus, recent findings show that cortical neurons that process tactile inputs are widely distributed across the neocortex. Since cortical neurons project back to the thalamus, the latter observation would suggest that thalamic neurons could contain information about tactile inputs, in principle regardless of where in the thalamus they are located. Here we use a previously introduced electrotactile interface for producing sets of highly reproducible tactile afferent spatiotemporal activation patterns from the tip of digit 2 and record neurons throughout widespread parts of the thalamus of the anesthetized rat. We find that a majority of thalamic neurons, regardless of location, respond to single pulse tactile inputs and generate spike responses to such tactile stimulation patterns that can be used to identify which of the inputs that was provided, at above-chance decoding performance levels. Thalamic neurons with short response latency times, compatible with a direct tactile afferent input via the cuneate nucleus, were typically among the best decoders. Thalamic neurons with longer response latency times as a rule were also found to be able to decode the digit 2 inputs, though typically at a lower decoding performance than the thalamic neurons with presumed direct cuneate inputs. These findings provide support for that tactile information arising from any specific skin area is widely available in the thalamocortical circuitry.

## Introduction

Information about tactile inputs is ubiquitous in the rat cortical circuitry, including primary visual cortex as demonstrated for a set of tactile input patterns delivered to digit 2 (Enander et al., 2019). Also, in humans, an fMRI study showed that specific responses in the primary somatosensory cortex could be triggered by specific visual inputs (Smith and Goodale, 2015). As the cortex projects back to the thalamus, and not just to homonymous thalamic nuclei (Halassa and Sherman, 2019), but to diverse parts of the thalamus, it would seem a natural consequence that also in the thalamus there would be ubiquitous representation of tactile information. This possibility has not been explored in great detail, as the focus in thalamic recording studies typically lie in the study of the modality expected to be specific for the thalamic nuclei recorded from. A recent study (Allen et al., 2017), though, showed that visual input affected the responses to tactile whisker stimuli in the primary somatosensory thalamic nucleus, the ventral posteromedial nucleus (VPM), in the anesthetized rat. Further, Bieler et al. (2018) found tactile inputs to evoke field potential responses in the lateral geniculate nucleus (LGN). They also found that individual cells in the VPM can project directly to the primary visual cortex. Moreover, it has previously been reported that primary sensory thalamic nuclei project to more than one primary sensory cortical area (Henschke et al., 2015). These findings show that tactile inputs can reach wide cortical areas, and since these cortical areas project back to different primary thalamic regions, a wide region of the thalamus which may not receive direct cuneate nucleus input (Alloway et al., 1994; Bermejo et al., 2003) can potentially be reached by tactile inputs via longer pathways.

Such findings are not widely reported in the literature and a possible reason for that could be the limited sensitivity of the analysis methods by which the presence of information concerning ‘unexpected’ modalities could be detected. In this study we used highly reproducible spatiotemporal tactile activation patterns delivered to the digit 2 combined with a decoding analysis of neuronal spike responses which has previously proven to be a highly sensitive tool for exploring the presence of neural signals reporting on the quality of tactile inputs in various parts of the neocortex (Oddo et al., 2017; Genna et al., 2018; Enander and Jorntell, 2019; Enander et al., 2019; Wahlbom et al., 2019). We recorded from a variety of thalamic regions in the anesthetized rat and find that a majority of thalamic neurons recorded have a detectable tactile input and that the spike responses of these cells as a rule carry information about the quality of tactile inputs delivered to the same population of tactile afferents, confined to the ventral side of the second forepaw digit.

## Results

**FIGURE 1:**
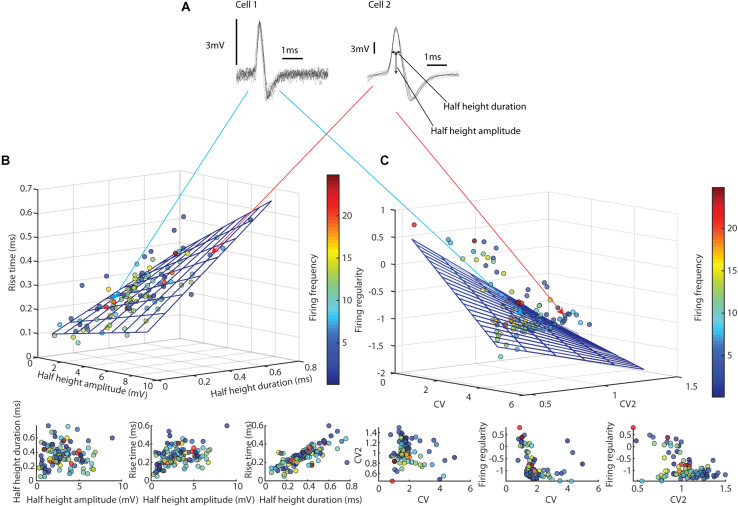
Spike firing metrics suggested that thalamic neurons formed a continuum, thus not offering a basis for subdividing our thalamic neurons into classes. **(A)** The spike shapes of two different thalamic neurons. Each example consists of 10 superimposed traces. **(B)** Relationship between spike shape parameters across all thalamic neurons recorded. The 3D plot at the top contains a multilinear regression overlaid on the data points. Below, each pairwise relationship is plotted separately. **(C)** Relationships between three different measures of spike firing regularity. The 3D plot on top contains a multilinear regression overlaid on the data points. Below, each pairwise relationship is shown separately.

**FIGURE 2:**
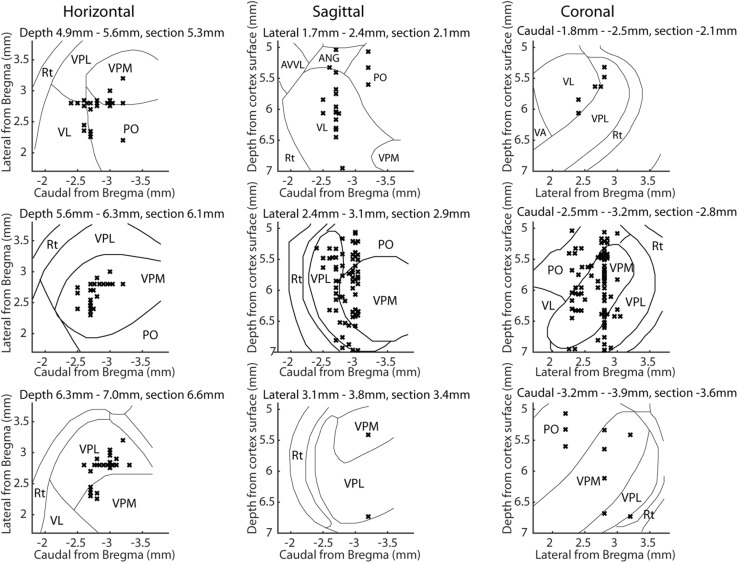
Location of neuronal recording sites in stereotaxic coordinates with the outline of the thalamic nuclei indicated. All thalamic neuron recording sites are shown in horizontal, sagittal and coronal view. For each viewing plane, the plane is split into three ranges and all recording sites within each range is shown. The ranges of each outline level are here presented as lower limit-upper limit the plane of the outline section illustrated; Paxinos and Watson, 2006). The left column shows the horizontal plane with top row: 4.9–5.3 mm (5.3 mm), middle row: 5.6–6.3 mm (6.1 mm) and bottom row: 6.3–7 mm (6.6 mm). In the same manner the middle column shows the sagittal plane with top row: 1.7–2.4 mm (2.1 mm), middle row 2.4–3.1 mm (2.9 mm) and bottom row: 3.1–3.8 mm (3.4 mm). Right column show the coronal range with top row_ –1.8 to –2.5 mm (–2.1 mm), middle row: –2.5 to –3.2 mm (–2.8 mm) and bottom row: –3.2 to –3.9 mm (–3.6 mm). Rt, Reticular thalamic nucleus; VPL, ventroposterior lateral nucleus; VPM, ventroposterior medial nucleus; VL, ventrolateral nucleus; PO, posterior complex; AVVL, anteroventral nucleus of thalamus ventrolateral part; ANG, anterior nuclear group.

We made extracellular unitary spike recordings (Figure 1A) from 109 thalamic neurons across the rostrocaudal and mediolateral extent of the thalamus (Figure 2). We analyzed these neurons with respect to their spike shapes, spike firing patterns, response latency times and decoding of a set of eight spatiotemporal tactile afferent activation patterns delivered to the ventral side of the distal digit 2.

### Spike Properties and Spike Firing Properties of Thalamic Neurons

As the neuronal population in the thalamic nuclei consists not only of thalamocortical projection neurons but also of local interneurons (Su et al., 2020), we first explored the spike shapes and the spike firing metrics of the recorded neurons for exploration of any possible distinct cluster formation separating these two tentative groups of neurons. The spike shape metrics (Figure 1B and Table 1) had no obvious tendency to form clusters, but instead seemed to form a continuum. A multilinear regression analysis showed tendencies toward a linear relationship [*F*(2, 106) = 93.0, *p* = 1.92 × 10^–29^, *r*^2^ = 0.73], which at least partly could be due to that there was a strong relationship between rise time and half height duration. Note also that the firing frequency varied widely between neurons (Table 1) but had no apparent impact on the spike shape metrics (Figure 1B).

**TABLE 1:** Population data of spike shape and spike firing metrics.

Spike/firing property	Mean ± SD
Half height amplitude	3.07 ± 1.80 mV
Half height duration	0.35 ± 0.15 ms
Rise time	0.27 ± 0.10 ms
Firing frequency	9.61 ± 5.26 Hz
CV	1.86 ± 0.73
CV2	1.05 ± 0.20
Firing regularity (log)	−0.90 ± 0.51

To quantify the spike firing regularity (Figure 1C), we used three measures; (1) the coefficient of variation (CV) of the interspike intervals (ISIs), which evaluates the spread of ISI values relative to the mean ISI for the entire spike train; (2) CV2, reported as an average value for each neuron, compares the relative difference between two adjacent ISIs (Holt et al., 1996), and (3) the “firing regularity” measure of Mochizuki et al. (2016), which describes the distribution of multiple ISIs. All these measures are dimensionless and reflect the spike train structure independently of the absolute firing rate of the neuron. Also in this case, there was little tendency for clustering (Figure 1C and Table 1), and again the distribution resembled a continuum, regardless of firing frequency. Here a multilinear regression analysis showed only weak tendencies toward a linear relationship [*F*(2, 106) = 28.0, *p* = 2.28 × 10^–13^, *r*^2^ = 0.44]. Based on the absence of any clear clusters for these metrics, we ascribe all our neuron recordings as being putative thalamic projection neurons, which is in line with an estimate of only about 1% of the neuron population being local inhibitory interneurons in the non-visual thalamic sensory relay nuclei of the rat (Arcelli et al., 1997).

### Decoding of Tactile Input Patterns

**FIGURE 3:**
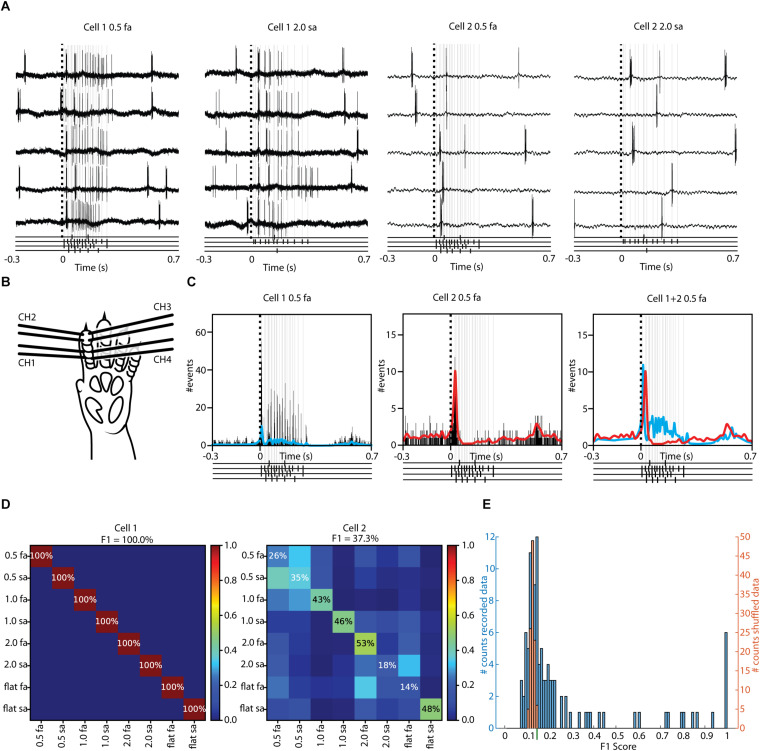
A majority of our recorded thalamic neurons had above chance decoding of tactile input. **(A)** Sample raw responses of two different neurons to two different stimulation patterns. Below each sample, the presented stimulation pattern is shown with black markers with light gray lines extending upwards. **(B)** Schematic of the rat forepaw showing the location of the four pairs of electrotactile stimulation electrodes (Supplementary Figure 1 illustrates the full set of stimulation patterns). **(C)** Peristimulus Time Histograms (PSTHs) and Kernel Density Estimations (KDEs) of all of the responses to one of the stimulation patterns for the two sample cells. The KDEs of the two cells are shown superimposed and normalized in the diagram to the right. Below each PSTH/KDE the presented stimulation pattern is shown with black markers with light gray lines extending upwards. **(D)** Confusion matrices of the decoding performance for the two sample cells across all eight stimulation patterns. **(E)** Decoding performance for the entire population of recorded neurons (blue bars). Orange bars show the corresponding distribution of the decoding following shuffling of the stimulation pattern labels. Green bar below the *x*-axis corresponds to the mean plus 2 SD of the shuffled data, which was the decision boundary for counting a thalamic neuron as being an above-chance decoder.

We next quantified the specificity of the spike responses to a fixed set of eight spatiotemporal patterns of activation of skin afferents in the ventral side of distal digit 2 [see section “Materials and Methods” for details, and furthermore the stimulation patterns are described graphically in Supplementary Figure 1 and in greater detail in Oddo et al. (2017); also Genna et al. (2018) and Enander et al. (2019)]. Figure 3A illustrates a set of raw spike responses to two of the stimulation patterns in two different cells. It can be noted that the dynamics of the overall activity differed substantially between the two neurons. It can also be noted that the responses to repeated applications of the same stimulation pattern varied, sometimes greatly, for the same neuron. Figure 3B shows a schematic displaying the placement of the stimulation electrodes used to create the spatiotemporal pattern of skin afferent activation. Displayed as peristimulus time histograms (PSTHs), the differences between the responses of the two neurons were more easily estimated (Figure 3C). As an alternative to the PSTH, we also overlaid a Kernel Density Estimation (KDE) (Shimazaki and Shinomoto, 2010) of the spike response (solid lines in Figure 3C), which further clarified the difference between the responses of the two neurons to the same stimulation pattern.

The differences between responses in the same neuron to each of the eight stimulation patterns were quantified using a decoding analysis similar to previous publications (Enander and Jorntell, 2019; Enander et al., 2019; Wahlbom et al., 2019). Using PCA of the individual spike responses to each stimulation pattern, the position in high-dimensional PC space was defined and related to the positions of other responses evoked by the same and by the other stimulation patterns using kNN classification. Figure 3D reports the decoding performance, measured as F1-score, for the two illustrated neurons. Cell#1 was clearly better at reporting correctly which stimulation pattern was delivered (100% F1 score, every single response for all eight stimulation patterns was correctly classified) than Cell#2 (37.3% F1 score). In fact, across all neurons previously recorded in the S1 neocortex (Oddo et al., 2017; Enander and Jorntell, 2019; Wahlbom et al., 2019) using the exact same method, we have never observed such high decoding accuracy as 100%. Across the whole population of recorded thalamic neurons, there were no less than 6 thalamic neurons with in principle perfect decoding, but otherwise the decoding performance varied greatly (Figure 3E). It should be noted that in this type of analysis, pure noise for eight stimulation patterns would result in a reported decoding performance of 12.5% (1/8), which would thus be the objective threshold for ascribing a minimal level of decoding performance to any single cell. Here, we instead used the existing responses of each neuron and shuffled their stimulation pattern labels in order to obtain a baseline for above-chance decoding performance for each neuron separately (Figure 3E, orange bars). The resulting mean decoding performance for the shuffled responses fell very closely to the theoretical 12.5% limit. However, the SD of the decoding for the shuffled data was non-negligible, and we therefore defined a limit of mean + 2 SD (12.42% + 2^∗^0.85% = 14.12%, approximately indicated in Figure 3E by the green bar below the *x*-axis) above which level a thalamic neuron was counted as being positively decoding the tactile afferent input patterns. According to this criterion, 65 of 109, or 59.6%, of the recorded thalamic neurons decoded the tactile input patterns. A one-sample Kolmogorov-Smirnov test combined with visual inspection was used to determine that the distributions for the decoding performance and the shuffled decoding performance was not normally distributed (for the F1-score, non-shuffled data, the *p*-value was 1.46 × 10^–27^, whereas for the shuffled data the *p*-value was 6.36 × 10^–29^), thus a non-parametric comparison was performed. A Kruskal-Wallis test rejects the null hypothesis that the two data samples came from the same distribution at a 1% significance level (*H* = 33.65, 1 d.f., *p* = 6.6 × 10^–9^). The mean decoding performance and SD for the recorded and shuffled data is presented in Table 2.

**TABLE 2:** Decoding performance across the population of thalamic neurons, compared with shuffled data.

Mean F1-score ± SD	0.2601 ± 0.2556
Mean shuffled F1-score ± SD	0.1242 ± 0.0085

### Response Latency Times

**FIGURE 4:**
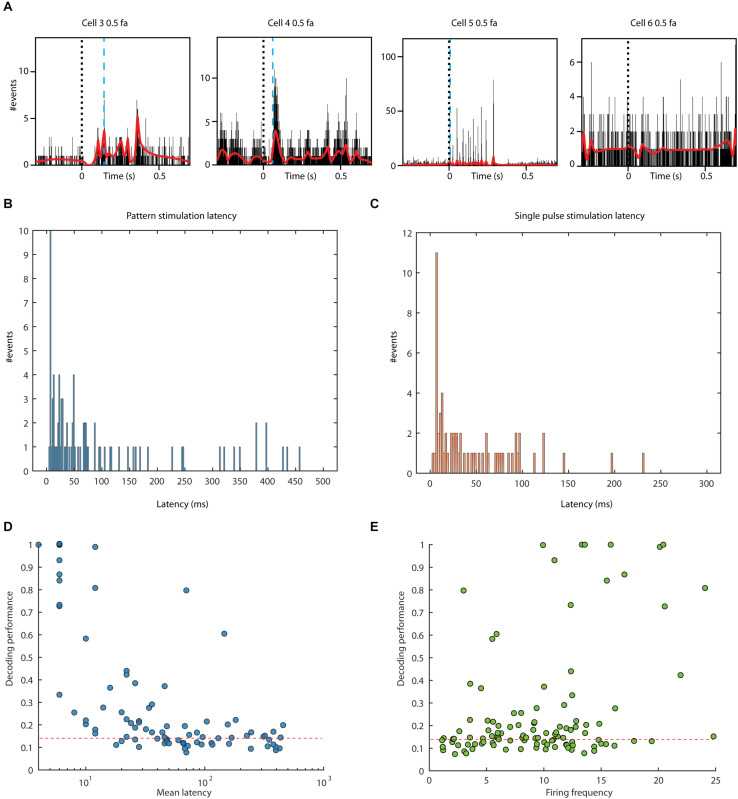
Response latency times and their relationship to decoding performance. **(A)** PSTHs and KDEs of the responses to one stimulation pattern in four sample cells. The blue dashed line indicates the estimated response onset latency time in each case (note that the response latency time for each neuron was calculated using the responses from all eight stimulation patterns). No response latency time could be identified for the neuron to the far right. **(B)** All detected response latency times for neurons during stimulation patterns. **(C)** All detected response latency times based on responses evoked by single pulse stimulation. **(D)** Relationship between decoding performance (F1-score) and the logarithm of the response latency time for pattern stimulation. Dashed red line indicates the decoding level threshold defined in Figure 3E. **(E)** Relationship between the decoding performance and the average firing frequency.

Displayed as PSTHs, it was again clear that different thalamic neurons could vary widely in the shape of the response they produced to individual stimulation patterns (Figure 4A). These PSTHs, were also used to calculate the response latency times (Figure 4A, dashed blue lines). A response latency time could not be identified for all thalamic neurons according to the criteria used (section “Materials and Methods”). However, when the response latency time of a neuron was extracted from the responses evoked by the stimulation patterns, 85/109 (78.0%) neurons had an identified response latency time (Figure 4B). When the latency times were instead based on the responses to isolated single pulse stimulations, 65/109 (59.6%) neurons had an identified response latency time (Figure 4C), the difference to the former being explicable by the fact that the stimulation patterns consisted of several stimulation pulses in sequence hence increasing the chance of detecting a response. There was a relationship between decoding performance and response latency time, in particular for neurons with the shortest response latency times (Figure 4D). A similar plot for the decoding performance against firing frequency revealed no clear relationship (Figure 4E).

The latency times calculated from the responses evoked by the stimulation patterns were obtained using a bin width of 2 ms (section “Materials and Methods”). Changing the bin width to 5 ms for this calculation, we instead obtained 76 neurons with an identified response latency time, whereas a bin width of 10 ms yielded 66 neurons with an identified response latency time. The fact that these times varied depending on the bin width could be explained by the higher threshold when using the longer bin widths, since the requirement was two consecutive bins being above two SD of the background activity regardless of bin width.

A one-sample Kolmogorov-Smirnov test combined with visual inspection showed that none of the observed latency distributions were normally distributed (stimulation pattern *p* = 3.4 × 10^–76^, single pulse *p* = 7.1 × 10^–57^). A Kruskal-Wallis test did not reject the null hypothesis that the data samples came from the same distribution at a 1% significance level (*H* = 4.86, 1 d.f., *p* = 2.7 × 10^–2^).

### Location of Decoding Performance and Response Latency Times

**FIGURE 5:**
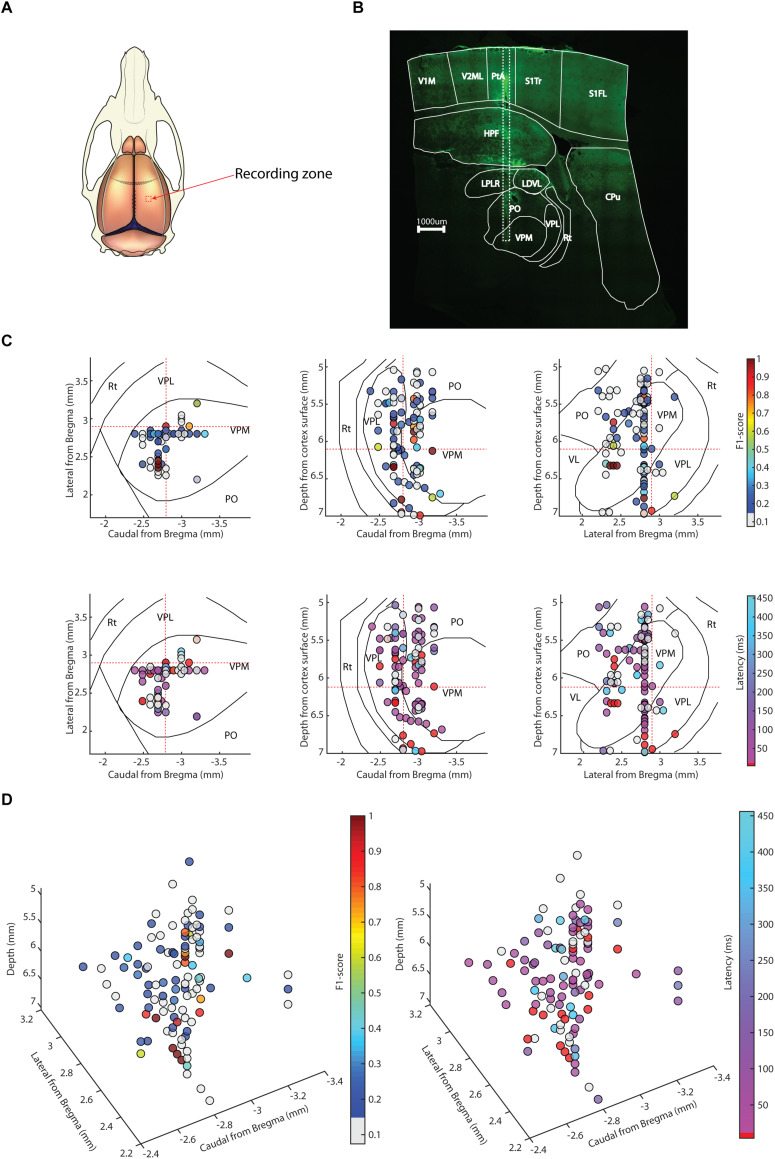
Relationships between location and decoding level and response latency time. **(A)** Schematic of the recording area from a dorsal view. **(B)** Sagittal section of the brain (with the thalamic nuclei outlined according to Paxinos and Watson, 2006) illustrating one electrode track (white dotted box). **(C)** 3D location of thalamic neurons visualized in three viewing directions, mapped onto one plane each (horizontal 6.3 mm, sagittal 2.9 mm and coronal –2.8 mm; more precise locations displayed in Figure 2) with color coded decoding performance (top row of plots) and response latency times (bottom row of plots). Response latency times of 10 ms or lower have been colored red. Dotted red lines in each plot indicate the stereotactic planes in the other two plots, for reference. **(D)** Similar display as in **(C)** but as 3D projections, with decoding performance shown to the left and response latency times to the right. Note that for visualization, multiple neurons recorded during the same recording session and electrode have been shifted 40 μm in the mediolateral axis, and neurons recorded very closely to each other according to stereotaxic coordinates, but in different sessions, have been shifted 40 μm in the rostrocaudal axis. V1, primary visual cortex; V2, secondary visual cortex; HPF, hippocampal formation; PtA, parietal area; S1, primary somatosensory cortex; CPu, caudate putamen (striatum); LPLR, lateroposero laterorostral nucleus; LDVL, laterodorsal ventrolateral nucleus.

We also explored whether there was a relationship between the location within the thalamus of the recorded neuron and its decoding performance or its response latency time (Figure 5). In brief, there was no strong relationship as estimated from stereotactic location, where Figure 5B illustrates a sample track that could be histologically verified to be in the same location as that indicated by the stereotaxic coordinates. A total of five recording tracks were identified histologically in five separate animals, in which the observed anterioposterior deviations of the recording location compared to that expected from the stereotaxic coordinate of the track amounted to an error of 0.24 +/− 0.21 mm (mean+/−SD; the maximum deviation was 0.5 mm). Hence, the stereotactic locations used for the displays in Figures 5C,D reasonably correctly reflected actual recording positions and hence a large proportion of the thalamus was recorded from (see also Figure 2). In addition, in 8 of the electrode tracks we recorded two or more neurons at a dorsoventral separation of >=1.0 mm (max neuron separation in one track was 1.51 mm), i.e., a distance spanning at least two separate thalamic nuclei. Thus, neurons with high decoding performance and relatively short response latency times were located throughout the dorsoventral, rostrocaudal and mediolateral stereotactic axes of the thalamus, as visualized in Figures 5C,D using 2D and 3D plots. (Note that in each plot, a 3D distribution of recording sites is mapped to a single 2D plane for each viewing direction. Figure 2 shows the locations of the same recording sites but instead maps the recording sites on three planes for each viewing direction, which gives a more accurate reflection of the thalamic nucleus in which a recording was made). Figures 5C,D show that there was possibly a tendency for a higher decoding performance among neurons in the ventral part of VPL, which has been identified as the main thalamic relay for cutaneous input from the forepaw skin (Francis et al., 2008; Li et al., 2014; Uemura et al., 2020). The response latency times followed a similar pattern of distribution.

## Discussion

We found that putative thalamic projection neurons distributed throughout all parts of the thalamus that we recorded from could decode tactile input patterns delivered to distal digit 2. Thalamic neurons with the shortest response latency times had a clear tendency to decode the tactile input patterns with the highest accuracy (Figure 4D). It is likely that some of these thalamic neurons received monosynaptic tactile input from the cuneate nucleus. All of the other thalamic neurons, which provided above chance decoding of the tactile input patterns, received tactile input at longer response latency times, which indicates that they received the tactile input via longer pathways. Such indirect pathways could for example include the neocortex, where at least minimal levels of neuronal decoding accuracy for tactile input patterns are widely present throughout various areas of cortex, including primary visual cortex (Enander et al., 2019). Indirect pathways could in addition to the cortex include the brain stem, the superior colliculus or the cerebellum (see below). Although approximately 40% of the recorded thalamic neurons did not exceed the set decoding threshold this is not necessarily definitive proof that they do not represent tactile information from digit 2. Weaker inputs could possibly have been found for example if we had been able to use more than 100 repetitions per tactile input pattern.

One reason that we found much more widespread representations of sensory information in the thalamic cells compared to previous reports, which tended to focus on thalamic nuclear specificity of sensory modalities (for example Brecht and Sakmann (2002)), is likely due to the type of input used. These spatiotemporal patterns of tactile afferent activation are highly reproducible and evolve in space and time in a way that creates a richly resolvable input (Oddo et al., 2017), which for this type of analysis offers an advantage compared to single shock stimuli or spatiotemporally highly reduced inputs. Detection of input, and input specificity, in the brain *in vivo* is typically achieved against a background of spontaneous activity, which in the analysis equals noise. Against this background, it is crucial to find a type of input that provides the possibility to separate signal from noise—but it is given that part of the signal will always be concealed by this noise and the experimental approach essentially becomes a matter of exposing as much as possible of the tip of the iceberg of the underlying signal. Whereas our approach is designed to overcome that problem, it still to some extent suffers from it. For example, a much higher number of repetitions of these eight input patterns would likely have exposed the tip of the iceberg among an even higher number of thalamic neurons. Hence, we believe that given sensitive enough methods, all thalamic neurons we recorded could well have been found to decode tactile input patterns from digit 2.

We made our electrode tracks according to stereotaxic coordinates with the aim to cover essentially the full rostrocaudal extent of the thalamus (although the medial thalamic nuclei were not covered) across the experimental series, and in a number of tracks we made at least two separate recordings separated by 1.0 mm or more dorsoventrally, i.e., spanning two or more thalamic nuclei. Identification of the thalamic neurons was also made using the response latency times, where any response recorded at 10 ms or below was considered to be indicative of a neuron receiving direct cuneothalamic input (Francis et al., 2008). However, only 8/109 recorded units had such short response latency times. For the rest of the units, the cutaneous input appeared to involve longer pathways, presumably primarily thalamo-cortico-thalamic, longer cortico-cortico-thalamic or brainstem pathways.

From the literature, units with direct cuneo-thalamic inputs from digit 2 would be expected to be primarily localized to a small part of VPL (Villanueva et al., 1998), adjacent to the VPM (Francis et al., 2008; Li et al., 2014; Uemura et al., 2020). However, the cuneate has also been observed to project to the posterior thalamic nucleus, although this observation applies to parts of the cuneate in which afferent with high precision information from digit 2 may possibly not exist (Berkley et al., 1986). In terms of brainstem pathways, the cuneate output has a high number of potential indirect routes (Loutit et al., 2021) to the thalamus, including the superior colliculus (Bezdudnaya and Castro-Alamancos, 2011; Gharaei et al., 2020). The cerebellar nuclei can be provided with cutaneous input via spinal interneurons and the lateral reticular nucleus (Bengtsson and Jorntell, 2014; Jorntell, 2017), and has powerful input to the motor thalamus (VL) (Jorntell and Ekerot, 1999), which is a potential explanation for the decoding of tactile digit 2 information we observed in this part of the thalamus.

In addition to the decoding performance analysis, we also analyzed the response latency times, where the presence of a defined latency time was a strong indication that the neuron did receive tactile input that came in addition to their decoding performance level. In this case, we could estimate that 76/109 (69.7%) of the neurons had detectable tactile input.

Ubiquitous thalamic representations of the quality of tactile input patterns is naturally in line with previous observations that such representations are also ubiquitous across cortical areas (Enander et al., 2019). They are also in line with that S1 receives direct input from multiple primary sensory thalamic nuclei, and that other primary sensory cortical areas receive input from non-homonymous primary sensory thalamic nuclei (Henschke et al., 2015) (also demonstrated for a VPL neuron projecting to V1, by Bieler et al. (2018)). In addition, it also fits well with observations that layer V neurons can provide input to thalamic nuclei other than the ones believed to primarily supply that cortical region (Halassa and Sherman, 2019). Furthermore, a subset of whisker-responsive neurons in VPM was also found to respond to visual input (Allen et al., 2017). Another potential contributing explanation for our observations would be if cuneate inputs terminate outside VPL, as discussed above, rather than having an exclusive projection to an assumed digit area in the VPL (Francis et al., 2008; Li et al., 2014; Uemura et al., 2020). Hence, whereas many observations and open alternatives support the basic finding of widespread thalamic representations of tactile inputs that we made here, a unique contribution of our study was that the thalamic neurons moreover has information about the detailed quality of the tactile stimulus to digit 2. Naturally, we do not expect such widespread thalamic representations to be a property that would exclusive apply to input from digit 2, or even the tactile sense, but likely represent a general principle for all sensory information as well as information being more internal to the neocortical circuitry.

## Materials and Methods

### Surgical Procedures

Adult male Sprague-Dawley rats (*N* = 29, weight 308–364 g) were prepared and maintained under anesthesia with a ketamine and xylazine mixture (20:1). Following isofluorane sedation (2% for 30–60 s), anesthesia was induced via an i.p. injection (40 mg/kg of ketamine, 2 mg/kg of xylazine) and maintenance was administered through an intravenous catheter inserted into the right femoral vein (∼5 mg/kg ketamine per hour with a continuous infusion). For recording sessions, the level of anesthesia was monitored with a surface electrocorticogram (ECoG) electrode placed in the vicinity of the recording area. The ECoG was characterized by irregular occurrences of sleep spindles, a sign of deep sleep (Niedermeyer and da Silva, 2005). The level of anesthesia was additionally characterized by an absence of withdrawal reflexes to noxious pinches to the hind paw. All animal experiment procedures in the present study were in accordance with institutional guidelines and were approved in advance by the Local Animal Ethics Committee of Lund, Sweden (permit ID M118–13).

### Recordings

All recording tracks were aimed to the thalamus according to the stereotaxic coordinates defined by Paxinos and Watson (2006). The location of the bregma was determined to be located at the point where the coronal and sagittal sutures crossed, and a flat elevation of the skull was ensured by placing bregma and lambda at the same relative height. According to these criteria all our recordings were performed in the thalamus.

Neurons were recorded with patch clamp pipettes extracellularly in the loose patch recording mode using the EPC 800 Patch Clamp Amplifier (HEKA, Lambrecht, Germany) without any applied filters. Patch clamp pipettes were pulled from borosilicate glass capillaries to 6–15M Ohm using a Sutter Instruments (Novato, CA) P-97 horizontal puller. The composition of the electrolyte solution in the patch pipettes was (in mM) potassium-gluconate (135), HEPES (10), KCl (6.0), Mg-ATP (2), EGTA (10). The solution was titrated to 7.35–7.40 using 1M KOH. In order to find neurons, the electrode was advanced with a stepping motor and the recording depths were tracked. The stimulation electrodes located in the skin of digit 2 were repeatedly activated at 0.3 s intervals and the characteristic evoked field potentials and occasional neuron spike recordings of the cortex and the hippocampus could be followed as we approached the thalamus. Once located in the dorsal part of the thalamus, the recording electrode was more slowly advanced (approximately 0.3–1.0 μm per second) while the same skin stimulation was active, and any spike activity encountered was taken as an indication of a neuron. The advancement then stopped, and attempts to isolate a single neuron were made. On successful isolation, a standard protocol of artificial tactile stimulation patterns (see below) was commenced. All data was digitized at 100 kHz using CED 1401 mk2 hardware and Spike2 software (Cambridge Electronics Devices, CED, Cambridge, United Kingdom).

### Tactile Afferent Stimulations

The recordings were made in a set of experiments similar to those in Oddo et al. (2017), where four pairs of stimulation electrodes (made of stainless steel insect pins of size 000, diameter 0.25 mm, with etched tips) were inserted into the volar side of the second digit of the contralateral forepaw. These stimulation electrodes delivered 0.5 mA pulses lasting 0.14 ms. As a standard protocol, the animal was then episodically presented with repeatable spatiotemporal patterns, reminiscent of the activation of primary afferents when touching objects with four different curvatures (in total eight patterns, named 0.5 fa, 0.5 sa, 1.0 fa, 1.0 sa, 2.0 fa, 2.0 sa, flat fa and flat sa), as described in Oddo et al. (2017). The eight spatiotemporal stimulation patterns were delivered in a pre-defined random order, where the stimulation patterns lasted for less than 340 ms and the consecutive deliveries of the stimulation patterns were separated by 1.8 s. In this relaxation phase, the firing activity of the neuron was then free from external inputs. Each pattern was delivered 100 times. The digit was also presented with single pulse stimulations, where only one pair of stimulation electrodes at a time were used to provide stimulation, 100 times for each electrode pair.

### Post Processing

The signal was imported from Spike2 to MATLAB (R2018b, The Mathworks, Inc.), where it was low-pass filtered using a moving average over 50 μs, i.e., a width of 5 samples. Cellular spikes were identified from the signal using tailored template matching routines with manually constructed templates. Each spike template was adapted to identify the same spike in all parts of the recording, as verified by visual inspection of a high number of random raw recording traces (visualized in time-voltage diagrams with a duration of 50–300 ms) in the beginning, the middle and the end of the recording.

### Spike Shape Analysis

All recorded spike shapes of a neuron were overlaid and a mean spike shape was created. From this shape, the spike amplitude was calculated from the inflection point to the maximum peak value. The rise time was calculated as the time between 10% of the spike amplitude and the maximum spike amplitude. The half-height amplitude was calculated as half of the spike amplitude, and the half height duration was the time between when the half height amplitude was crossed on the rising and falling phase of the mean spike shape.

### Firing Behavior Analysis

Three different methods were used to evaluate the spike firing regularity of the recorded neurons. In all three cases the measurement was based on the interspike intervals (ISI). The coefficient of variation CV was calculated as

(1) $$CV=\frac{\sigma_{ISI}}{\mu_{ISI}}$$

where σ_*ISI*_ is the SD of the ISIs of the neuron and μ_*ISI*_ is the mean ISI.

The CV2 of a neuron compares two adjacent ISIs and was calculated as

(2) $$CV2=\frac{2\left|ISI_{i+1}-ISI_{i}\right|}{ISI_{i+1}+ISI_{i}}\;$$

where ISI_*i*_ is the ith ISI and ISI_*i*__+__1_ the following ISI, and was presented as the average of all CV2s of a neuron. The last measure used was the firing regularity, as shown in Mochizuki et al. (2016). A gamma distribution was fitted to the distribution of the ISIs of a neuron using the gamfit function in MATLAB (R2018b, The Mathworks, Inc.) and a maximum likelihood estimate for the shape factor was extracted. The firing regularity is then presented as the logarithm of the shape factor. Lastly the average firing frequency of a neuron was calculated by dividing the number of recorded spikes with the recording duration.

### Decoding Performance

In order to evaluate the neuronal response to the electrotactile stimulation of the second digit we used a modified version of a previously published method (Oddo et al., 2017). The method uses bootstrapping of the neuronal data, principal component analysis (PCA) and k-nearest neighbor (kNN) classification in order to evaluate how well a neuron can differentiate between different spatiotemporal tactile inputs, reported as the neuron’s F1-score for the classification task, here called decoding performance. A more detailed description of the method is described below.

(i) We used an exponential kernel with a characteristic time of 5 ms to convolve the spike trains evoked by each stimulation presentation into continuous functions, following 1,000 ms after the start of the stimulation.
(ii) The convolved responses were randomly assigned to one of two groups, half into a training set and the other half into a test set. These two data sets were handled separately for the remainder of the analysis.
(iii) Bootstrapping was used to resample each data set 200 times and PCA was used on the training set in order to determine the N principal components (PCs) that were required to explain 95% of the variance observed in the bootstrapped data.
(iv) The scalar product for each bootstrapped response and N PCs was then computed using the least squares method. From this result, each bootstrapped response could be placed in an N-dimensional space.
(v) The last step of the analysis was to use the kNN-classification procedure to decode the stimuli from each bootstrapped response. For each response in the bootstrapped test set the nine closest responses in the training set were identified using a Euclidian distance calculation in the N-dimensional space. A test response was classified as belonging to the same pattern that had a relative majority out of the nine closest responses from the training set.
(vi) These five steps were repeated 50 times, with each repetition having a new random division of the convolved responses into a training and test set. The data from these 50 iterations was combined and an average confusion matrix was obtained.

From this confusion matrix the precision and recall of the classifier was calculated as

(3) $$Precision=\frac{TP}{TP+FP}\;$$

(4) $$Recall=\frac{TP}{TP+FN}\;$$

Were TP are the True Positives, FP the False Positives and FN the False Negatives. From these two parameters the F1-score, which we use as a measure of decoding performance, was calculated as their harmonic mean,

(5) $$F1=2\times \frac{Precision\times Recall}{Precision+Recall}$$

The full method was repeated a second time, but with the spike train responses shuffled with respect to the presented stimulation pattern before a split into training and test set was made. The resulting mean and SD of the F1 score for the entire population of neurons was used to create a chance F1-score limit defined as the mean plus two SD. If a neuron’s F1-score for the non-shuffled data set was above this limit it was counted as being able to decode the tactile information.

The bootstrapping used in this method is described in further detail here. First, the convolved responses were grouped by stimulation pattern, then a new sample of N responses were taken from this population using sampling with replacement, where N was equal to the number of available responses. The sum of these responses was stored as a bootstrapped response.

### Latency Analysis

The neuronal response latency times were defined as the time between the onset of a stimulus, either pattern stimulation or single pulse stimulation, and the time point were two consecutive bins of 2 ms in a peristimulus time histogram (PSTH) of the stimulated activity exceeded the mean + 2 SD. The SD was calculated from a PSTH of the spontaneous activity, according to the rate change method presented in Levakova et al. (2015). The PSTH of the activity evoked by the pattern stimulation was based on all 800 trials, from the onset of the stimulation and for a duration of 500 ms, divided into bins of 2 ms. In a similar manner, the 400 trials of single pulse stimulations were combined into a PSTH lasting 300 ms. All data used for the latency analysis was normalized based on the number of trials for each neuron and PSTH type (pattern or single pulse stimulation).

### Histology

In five experiments, the patch pipette electrolyte solution was mixed with Neurobiotin Tracer (Vector Laboratories) in order to stain recorded neurons or the tracks made to reach them. The animals were kept under general anesthesia and then transcardially perfused using 100 ml phosphate buffered saline (PBS) followed by 75 ml 4% paraformaldehyde (PFA) solution. The brains were then removed and post-fixed in 4% PFA solution for 48–72 h and stored in PBS. Before sectioning, the brain was submerged in 25% sucrose in PBS for 48 h. Sixty micrometer sections were then cut using a cryostat-microtome and the sections were stained with Streptavidin conjugated to Alexa Flour 488 (Molecular Probes Inc.). A confocal microscope combined with a fluorescence microscope was then used to identify any stained neurons and electrode tracks. This was used together with the atlas by Paxinos and Watson (2006) in order to determine the location of the recorded neuron, which was compared with the location according to stereotaxic coordinates.

## Data Availability Statement

The data is available on https://figshare.com/collections/Widespread_decoding_of_tactile_input_patterns_among_thalamic_neurons/5291314.

## Ethics Statement

The animal study was reviewed and approved by the Local Animal Ethics Committee of Lund, Sweden (permit ID M118–13).

## Author Contributions

AW and HJ designed the experiments. AW performed all the experiments. AW, HJ, and JE designed and performed the analysis and wrote the manuscript. All authors contributed to the article and approved the submitted version.

## Conflict of Interest

The authors declare that the research was conducted in the absence of any commercial or financial relationships that could be construed as a potential conflict of interest.

## Supplementary Material

Supplementary Figure 1 **(A)** Schematic of the rat forepaw showing the placement of the four pairs of stimulation electrodes (color coded). **(B)** The eight stimulation patterns used as the tactile inputs. Each color corresponds to a stimulation channel as indicated in **(A)**.
