# Supplementary figures and images for: Widespread Decoding of Tactile Input Patterns Among Thalamic Neurons
Source: Front Syst Neurosci. 2021 Feb 16;15:640085. doi: 10.3389/fnsys.2021.640085 (PMC7921320; doi:10.3389/fnsys.2021.640085)

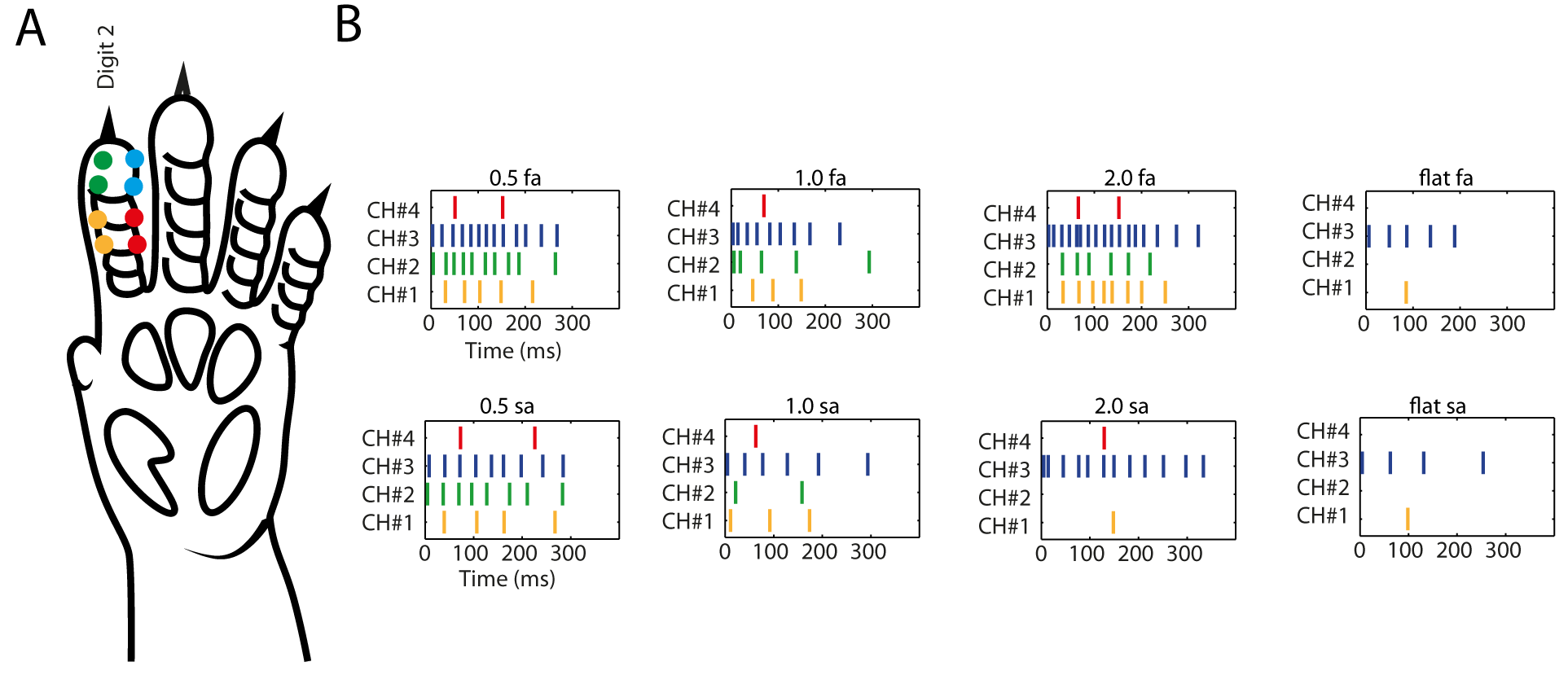

Supplement: Supplementary Figure 1 — (A) Schematic of the rat forepaw showing the placement of the four pairs of stimulation electrodes (color coded). (B) The eight stimulation patterns used as the tactile inputs. Each color corresponds to a stimulation channel as indicated in (A). [file Image_1.TIF]
